# Clinical Evaluation of Giomer-Based and Nanohybrid Restorative Materials in Non-Carious Cervical Lesion Class V Restorations over 72 Months: A Randomized, Split-Mouth Clinical Trial

**DOI:** 10.3390/dj14090603

**Published:** 2026-09-17

**Authors:** Adam Lowenstein, Carlos Fernando Mourão, Mabi L. Singh, Sarah E. Pagni, Ronald D. Perry, Gerard Kugel

**Affiliations:** 1 Department of Basic and Clinical Translational Sciences, Tufts University School of Dental Medicine, Boston, MA 02111, USA; carlos.mourao@tufts.edu; 2 Division of Oral Medicine, Department of Diagnostic Sciences, Tufts University School of Dental Medicine, 1 Kneeland St, Boston, MA 02111, USA; mabi_l.singh@tufts.edu; 3 Department of Public Health and Community Service, Tufts University School of Dental Medicine, 1 Kneeland St, Boston, MA 02111, USA; sarah.pagni@tufts.edu; 4 Department of Comprehensive Care, Tufts University School of Dental Medicine, 1 Kneeland St, Boston, MA 02111, USA; ronald.perry@tufts.edu; 5 College of Dentistry, University of Nebraska Medical Center, Omaha, NE 68198, USA; gkugel@unmc.edu

**Keywords:** restorative material, dental materials, operative dentistry, composite

## Abstract

**Background/Objectives:** This randomized, split-mouth clinical trial compared the 72-month clinical performance of a giomer-based restorative material (BEAUTIFIL II LS) and a nanohybrid restorative composite (Filtek Supreme Universal) in Class V non-carious cervical lesions (NCCLs). **Methods:** Forty-nine participants, each with two paired NCCLs, were enrolled (98 restorations) and received both materials under a standardized total-etch adhesive protocol. Clinical performance was assessed at 72 months using modified Hickel/FDI criteria across esthetic, functional, and biological domains, with between-material comparisons performed by paired nonparametric analysis (Wilcoxon signed-rank test) for complete paired observations. **Results:** Of the 49 participants enrolled, 26 attended the 72-month follow-up visit and 25 contributed evaluable data, yielding 44 evaluable restorations. No restoration was lost, and no statistically significant differences were detected between BEAUTIFIL II LS and Filtek Supreme Universal for any evaluated esthetic, functional, or biological criterion. Both materials showed acceptable clinical performance, with minor changes related mainly to marginal staining and marginal adaptation over time. **Conclusions:** BEAUTIFIL II LS and Filtek Supreme Universal showed similar long-term clinical performance in Class V non-carious cervical lesion restorations over 72 months. Within the limitations imposed by long-term attrition, material selection may be guided by clinical requirements, handling characteristics, and clinician preference.

## 1. Introduction

Class V restorations present a distinctive clinical challenge due to their anatomical location at the cervical margin and the complex mechanical environment they must withstand. Non-carious cervical lesions (NCCLs), characterized by loss of tooth structure at the cemento-enamel junction, are increasingly prevalent in modern dental practice [1]. These lesions demonstrate a multifactorial etiology involving occlusal stress concentration, mechanical abrasion, erosion, and abfraction, with occlusion-generated stresses creating tissue loss at the cervical region [1]. The clinical manifestations extend beyond structural compromise; patients frequently experience aesthetic concerns, dentin hypersensitivity, weakened tooth architecture, and increased susceptibility to secondary disease [2]. Compounding these challenges is the difficulty in achieving adequate mechanical retention, as the minimal healthy tooth structure available at the cervical margin limits traditional cavity preparation design [3].

Composite resin materials have become the standard of care for direct restoration of cervical lesions, offering superior aesthetic and mechanical properties compared to alternative materials [4]. However, success in Class V restorations depends critically on the selection of restorative systems that demonstrate both strong adhesive bonding and favorable mechanical characteristics, particularly adequate fracture toughness and elasticity [5]. Polymerization shrinkage remains a significant concern in resin composite restorations, with reported volumetric shrinkage values ranging from 1.9% to 13.5%, potentially creating internal stresses and compromising the marginal seal at the tooth-restoration interface [6].

Recent advances in restorative materials have yielded two divergent technological approaches. Giomer-based materials incorporate surface pre-reacted glass-ionomer (S-PRG) fillers, which release multiple therapeutic ions, including strontium, fluoride, silicate, borate, sodium, and aluminum, providing bioactive properties such as acid neutralization, remineralization promotion, and antimicrobial effects [7]. The S-PRG filler technology has demonstrated remarkable capacity to inhibit enamel demineralization and promote remineralization through continuous ion release [7]. Conversely, nanohybrid restorative composites utilize non-agglomerated nanofillers and optimized resin matrices designed to enhance esthetic properties, wear resistance, and polish retention [8]. Recent clinical evidence indicates that both giomers and nano-hybrid composites exhibit comparable clinical performance in various restorative applications, with studies demonstrating that giomer materials and nanohybrid resins achieve similar surface characteristics and esthetic outcomes while maintaining comparable retention and marginal adaptation [2].

The reduced polymerization shrinkage of giomer materials offers potential advantages over conventional composites by creating less residual stress at the tooth-restoration interface and potentially improving marginal seal integrity [9]. Furthermore, the remineralizing potential of materials containing S-PRG fillers through fluoride and ion release may provide enhanced caries prevention, particularly beneficial in populations with compromised salivary flow [9]. Despite these theoretical advantages, direct clinical comparisons of giomer-based and nanohybrid restorative composites in extended longitudinal studies remain limited, particularly in the cervical restoration environment, where biomechanical stresses are most pronounced.

The purpose of this 72-month randomized, split-mouth clinical trial was to provide clinicians with evidence-based information regarding the long-term clinical performance of a giomer-based restorative system (BEAUTIFIL II LS) compared to a nanohybrid restorative composite (Filtek Supreme Universal) when used to restore non-carious cervical lesions. By evaluating multiple clinical domains using standardized Hickel criteria, this investigation aimed to determine whether differences in material composition and filler technology translate into clinically meaningful differences in restoration durability and performance over an extended follow-up period. The null hypothesis was that no significant differences would be detected between the two restorative systems over the 72-month follow-up period.

## 2. Materials and Methods

### 2.1. Study Design, Setting, and Ethical Oversight

This manuscript reports the 72-month follow-up of a randomized, controlled, split-mouth clinical trial conducted at the Tufts University School of Dental Medicine (TUSDM) Research Clinic (Boston, MA, USA). The original trial was designed to compare two resin-based restorative materials placed in paired non-carious cervical lesions (NCCLs) within the same participant, thereby minimizing inter-individual variability.

The study was conducted in accordance with the Declaration of Helsinki and applicable national and institutional regulations. The parent protocol received approval from the TUSDM Institutional Review Board (IRB #12486; initial approval: 5 May 2017). The trial was prospectively registered at ClinicalTrials.gov (NCT03153969; registration date: 17 May 2017). The study remained under active IRB oversight and continuing review throughout the follow-up period, including the 72-month assessment.

**Figure 1:**
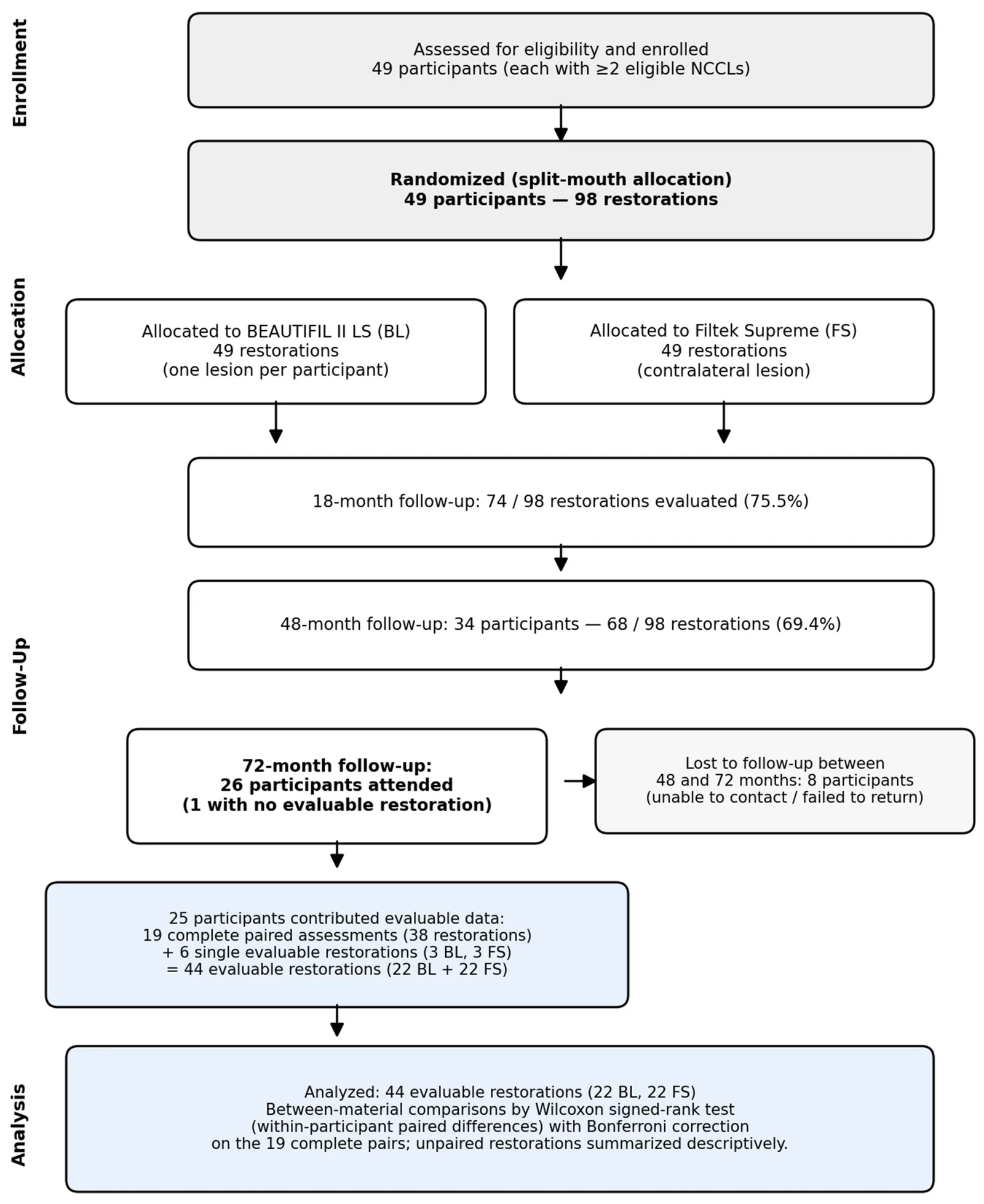
CONSORT flow diagram for the randomized split-mouth trial and long-term follow-up. Forty-nine participants with two eligible non-carious cervical lesions (NCCLs) were enrolled and randomized in a split-mouth allocation, such that each participant received both restorative materials (one lesion restored with BEAUTIFIL II LS [BL] and the contralateral lesion restored with 3M/ESPE Filtek Supreme Universal Restorative [FS]; 98 restorations total). Follow-up evaluations were conducted at 6, 18, 48, and 72 months. At 48 months, 34 participants were clinically assessed. At 72 months, 26 participants attended the follow-up examination, while 8 participants were lost to follow-up between 48 and 72 months due to inability to contact and/or failure to return for evaluation. At the 72-month visit, 1 participant had no evaluable restoration; of the remaining 25, 19 contributed complete paired assessments (both restorations evaluated) and 6 contributed a single evaluable restoration (3 BL, 3 FS). Analyses were planned to account for within-participant clustering and to include sensitivity analyses restricted to complete paired 72-month assessments. The 72-month visit thus yielded 44 evaluable restorations (38 from the 19 participants with complete paired assessments plus 6 single evaluable restorations; 22 BL and 22 FS).

All participants provided written informed consent prior to enrollment and agreed to longitudinal clinical evaluations. This report follows CONSORT principles for randomized trials, with additional transparency regarding long-term follow-up and missing outcome data (Figure 1) [10].

### 2.2. Sample Size Calculation

The sample size for the parent randomized, split-mouth trial was determined before enrollment, using the primary clinical outcome defined in the original protocol. The sample size calculation was based on prior clinical data for resin-based restorations in Class V non-carious cervical lesions [6,7], a paired split-mouth design, a two-sided significance level of 0.05, and a target of 80% statistical power for the primary follow-up endpoint of the parent trial [11]. On this basis, 49 participants, each contributing two eligible lesions (98 restorations at baseline), were enrolled. Because the present manuscript reports the 72-month follow-up of this trial, the long-term analysis was affected by participant attrition and should therefore be interpreted as a follow-up analysis rather than as a newly powered equivalence trial.

### 2.3. Participants, Eligibility Criteria, and Enrollment

Eligibility criteria were consistent with the parent trial. Participants were required to present at least two NCCLs in different teeth eligible for Class V restoration. Lesions had to permit placement of a minimum restorative thickness while maintaining the natural tooth contour, with the coronal margin located in enamel and a substantial dentin component (≥50%) within the lesion.

Key exclusion criteria included clinical or radiographic signs suggestive of pulpal or periapical pathology; non-vital teeth or teeth with prior endodontic treatment; lesions approaching pulp exposure based on radiographic assessment; pronounced dentin hypersensitivity at baseline that could compromise follow-up evaluation; advanced periodontal disease; and teeth serving as abutments for removable prostheses.

### 2.4. Randomization and Allocation (Split-Mouth Procedure)

Randomization was performed at the lesion level within each participant, assigning one NCCL to restorative material BEAUTIFIL II LS (BL; Shofu Inc., Kyoto, Japan) and the contralateral (paired) lesion to material 3M/ESPE Filtek Supreme Universal Restorative (FS; 3M/ESPE, St. Paul, MN, USA). This within-participant allocation ensured that each subject served as their own control. The randomization sequence was generated using a computerized spreadsheet tool (Microsoft Excel Version 16.89; Microsoft Corp., Redmond, WA, USA) by a team member not involved in clinical evaluations. Allocation was implemented at the time of restoration placement according to the sequence generated for each participant. Within each participant, the allocation determined which lesion received BL versus FS, with the contralateral lesion receiving the alternate material.

### 2.5. Restorative Materials

The test material, BEAUTIFIL II LS (Shofu Inc., Kyoto, Japan), is a nanohybrid composite containing surface pre-reacted glass-ionomer (S-PRG) filler with a polymer matrix composed of low-shrinkage urethane diacrylate, Bis-MPEPP, Bis-GMA, and TEGDMA. The bonding system used was BeautiBond (Shofu Inc., Kyoto, Japan), an acetone/water solvent-based system incorporating phosphonic acid and carboxylic acid monomers.

The control material, 3M/ESPE Filtek Supreme Universal Restorative (3M/ESPE, St. Paul, MN, USA), is a nanohybrid restorative composite formulated from Bis-GMA, UDMA, TEGDMA, and Bis-EMA(6), with non-agglomerated/non-aggregated silica and zirconia nanofillers. The bonding system used was Scotchbond™ Universal Adhesive (3M/ESPE, St. Paul, MN, USA), containing MDP phosphate monomer, dimethacrylate resins, HEMA, and Vitrebond™ copolymer.

### 2.6. Clinical Procedures and Standardization

Clinical procedures followed the same protocol used in the parent trial and the previously published follow-up reports. Briefly, NCCLs were managed conservatively with the goal of preserving sound enamel and dentin. Cavity preparation was performed using diamond burs (Brasseler USA, Savannah, GA, USA), followed by finishing with non-fluoride pumice. Isolation was achieved using Isolite (Zyris, Santa Barbara, CA, USA) or Mr. Thirsty (Zirc, Buffalo, MN, USA) in conjunction with cotton rolls as needed. A total-etch adhesive approach was applied for both materials: enamel and dentin surfaces were etched with 35–37% phosphoric acid for 15 s, rinsed thoroughly with water, and gently air-dried. BeautiBond was applied following the manufacturer’s instructions after phosphoric-acid etching for all BL restorations. Scotchbond™ Universal Adhesive was used in etch-and-rinse mode for all FS restorations. Both adhesives were actively applied for 20 s, air-dried for 5 s to evaporate solvents, and light-cured for 10 s according to the manufacturers’ instructions. Restorations were finished and polished using the Super Snap Rainbow Technique Kit (Shofu Inc., Kyoto, Japan). Clinical photographs were obtained at baseline and follow-up visits using the EyeSpecial C-II dental camera system (Shofu Inc., Kyoto, Japan) to document restoration condition.

Two calibrated clinicians placed the restorations at baseline. The operative steps, instruments, and materials were standardized across operators. No changes to restorative protocol, adhesive strategy, or finishing/polishing approach were introduced for the 72-month follow-up; the present report evaluates long-term clinical performance of the restorations originally placed at baseline.

### 2.7. Blinding, Examiner Calibration, and Quality Control

Participants were masked to material assignment at each lesion. Clinical evaluations were performed by examiners who were not involved in restoration placement and were masked to allocation. Prior to the beginning of the clinical trial, examiners underwent training and calibration on the scoring criteria. Calibration included independent scoring of a set of reference photographs/cases, followed by consensus discussion to standardize scoring. The calibration set included a representative sample of reference images/cases spanning the full range of modified Hickel scores, and scoring discrepancies were resolved through consensus discussion. Examiners were the same as those who performed the 48-month evaluations, ensuring continuity in assessment criteria.

To reduce measurement variability, efforts were made to maintain examiner continuity across visits; when the original examiner was unavailable, evaluation was performed by another calibrated, masked examiner using the same criteria.

### 2.8. Follow-Up Schedule and Ascertainment at 72 Months

The parent trial included a baseline visit (screening and restoration placement) and follow-up examinations at 6, 18, and 48 months. The present report adds a 72-month follow-up examination to assess long-term clinical outcomes. The 72-month visit was scheduled within a predefined window of 72 ± 2 months (70–74 months post-baseline) to accommodate participant availability while maintaining temporal consistency for outcome evaluation. The actual visit timing was recorded for each participant to characterize any variability in follow-up duration.

Participants who did not attend the 72-month visit were contacted using a standardized protocol: a minimum of six contact attempts were made over an 8-week period via telephone and email using the information provided at enrollment. Individuals who could not be reached or who did not return for the evaluation after these attempts were classified as lost to follow-up for the 72-month assessment.

### 2.9. Outcomes and Clinical Evaluation Criteria

Clinical performance was assessed using modified Hickel/FDI-based criteria [12] covering esthetic, functional, and biological domains. Outcomes included, but were not limited to: surface luster, surface staining, marginal staining, color match, anatomic form, fracture/retention, marginal adaptation, and patient-reported perception; and biological endpoints such as recurrent caries, tooth integrity, and adjacent mucosal condition. Scoring definitions followed the same grading framework applied in earlier follow-up reports (Table S1).

Restoration, retention, and failures were recorded at each scheduled follow-up visit. A restoration was considered a failure if it required replacement, was missing, or could not be clinically evaluated due to complete loss; partial defects managed without replacement were recorded according to the functional criteria and reflected in the relevant scores.

### 2.10. Handling of Attrition and Paired/Unpaired Observations (72-Month Follow-Up)

Long-term follow-up inevitably resulted in incomplete attendance at the 72-month visit. Of the 34 participants evaluated at 48 months, 26 attended the 72-month examination. Eight participants were classified as lost to follow-up between 48 and 72 months after unsuccessful contact attempts and failure to return for the scheduled evaluation.

Because the trial used a split-mouth design, the analytic unit remained the restoration, with clustering at the participant level. At 72 months, 26 participants attended; 1 had no evaluable restoration. Of the remaining 25, 19 contributed complete paired observations (both restorations assessed) and 6 contributed a single evaluable restoration (one side assessed and the contralateral restoration not available for clinical evaluation at that visit). Unpaired observations were retained to avoid discarding valid outcome data, and no contralateral outcomes were imputed.

To assess the potential for attrition-related bias, baseline characteristics and/or restoration status at the prior follow-up were compared between participants who attended the 72-month visit and those lost to follow-up. In addition, a prespecified sensitivity analysis was conducted restricting inference to the subset of participants with complete paired 72-month assessments (*n* = 19), preserving the within-participant comparison inherent to the split-mouth design.

### 2.11. Statistical Analysis

Statistical analyses were performed using Stata 17 (StataCorp LLC, College Station, TX, USA) and IBM SPSS Statistics 28 (IBM Corp., Armonk, NY, USA). Participant characteristics and clinical outcomes were summarized using descriptive statistics at each follow-up time point.

Given the ordinal nature of the modified Hickel/FDI-based criteria and the paired structure of the split-mouth design, between-material comparisons were performed using the Wilcoxon signed-rank test applied to within-participant paired differences (BL vs. FS) at each follow-up time point. The 6 participants who contributed only a single evaluable restoration at 72 months were included in the descriptive summaries of individual material performance but were excluded from the paired between-material comparisons, consistent with the requirements of the signed-rank test. Medians and interquartile ranges (IQR) were reported for each Hickel domain by material and visit. Mixed-effects ordinal regression, generalized estimating equations, material-by-time interaction terms, and model-based 95% confidence intervals were not used and are not reported.

For clinically relevant binary outcomes such as restoration loss/retention failure, failures were tabulated by material and follow-up time. Because exact failure dates were not available (failures were detected at scheduled visits rather than at the time of occurrence), time-to-event data were interval-censored. Retention was therefore summarized descriptively by material and visit; no inferential survival model or model-based between-material comparison was applied to these outcomes.

Handling of missing data: The primary analysis assumed that data were missing at random (MAR), conditional on observed covariates and prior outcomes. This assumption was supported by the comparison of baseline characteristics between completers and those lost to follow-up. No imputation was performed for missing outcome data; an available-case approach was used, with unpaired observations contributing to descriptive summaries of individual material performance but not to the paired between-material comparisons.

Sensitivity analyses included: (1) Complete paired subset analysis restricted to participants with both restorations evaluated at 72 months (*n* = 19), using within-participant paired comparisons consistent with the split-mouth design; (2) Attrition assessment comparing baseline characteristics and/or earlier follow-up status (e.g., 48-month scores/retention) between participants attending the 72-month visit (*n* = 26) and those lost to follow-up (*n* = 8).

All tests were two-sided. A *p*-value < 0.05 was considered statistically significant. When multiple pairwise comparisons were performed within a given Hickel domain across time points, Bonferroni correction was applied to control the family-wise error rate, consistent with prior publications from this trial series.

## 3. Results

This split-mouth study started with 49 subjects, each with two restorations placed under the established TUSDM standard of care, totaling 98 restorations at the baseline visit. At 18-month recall examinations, 74 (75.5%) restorations were evaluated. At the 48-month recall examination, 34 patients, totaling 68 (69.4%) restorations, were evaluated. At the 72-month recall examination, 26 participants attended; 1 had no evaluable restoration, and the remaining 25 contributed 44 evaluable restorations (44 of 98; 44.9%): 19 participants contributed complete paired assessments (38 restorations) and 6 contributed a single evaluable restoration (3 BL, 3 FS), yielding 22 BL and 22 FS restorations (Figure 1).

At 18-month follow-up, 39 of 43 (90.7%) restorations of BL were intact and acceptable, and 41 of 43 (95.3%) restorations of FS were intact and acceptable. After 48 months, 30 of 34 (88.2%) restorations of BL were intact and acceptable, and 32 of 34 (94.1%) restorations of FS were intact and acceptable. At 72 months, 22 BL and 22 FS restorations were evaluated. No restoration in either group was scored as lost or requiring replacement (Hickel fracture/retention score of 5); on the fracture/retention criterion, all 22 BL (100%) and all 22 FS (100%) were clinically acceptable (score ≤ 3). When acceptability was defined across all Hickel domains (every domain scored ≤3), 19 of 22 BL (86.4%) and 18 of 22 FS (81.8%) were rated clinically acceptable, with no statistically significant between-material difference. The demographic characteristics of participants who attended the 72-month follow-up are presented in Table 1.

**Figure 2:**
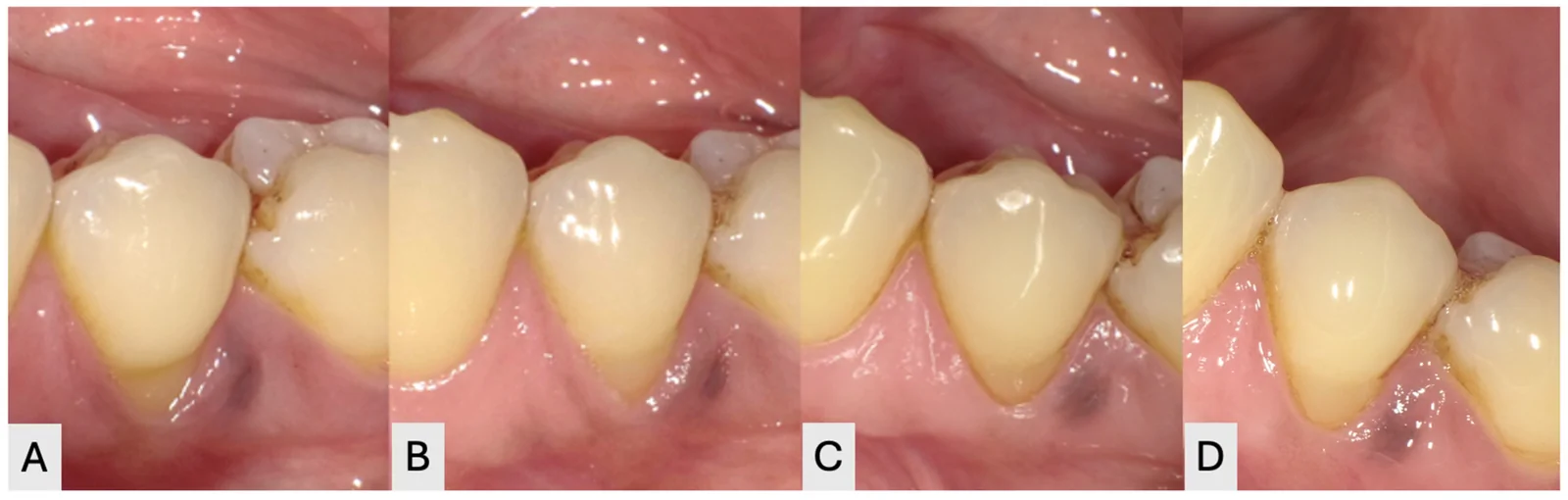
Clinical image of BL at (**A**) baseline, (**B**) 18 months, (**C**) 48 months, and (**D**) 72 months.

**Figure 3:**
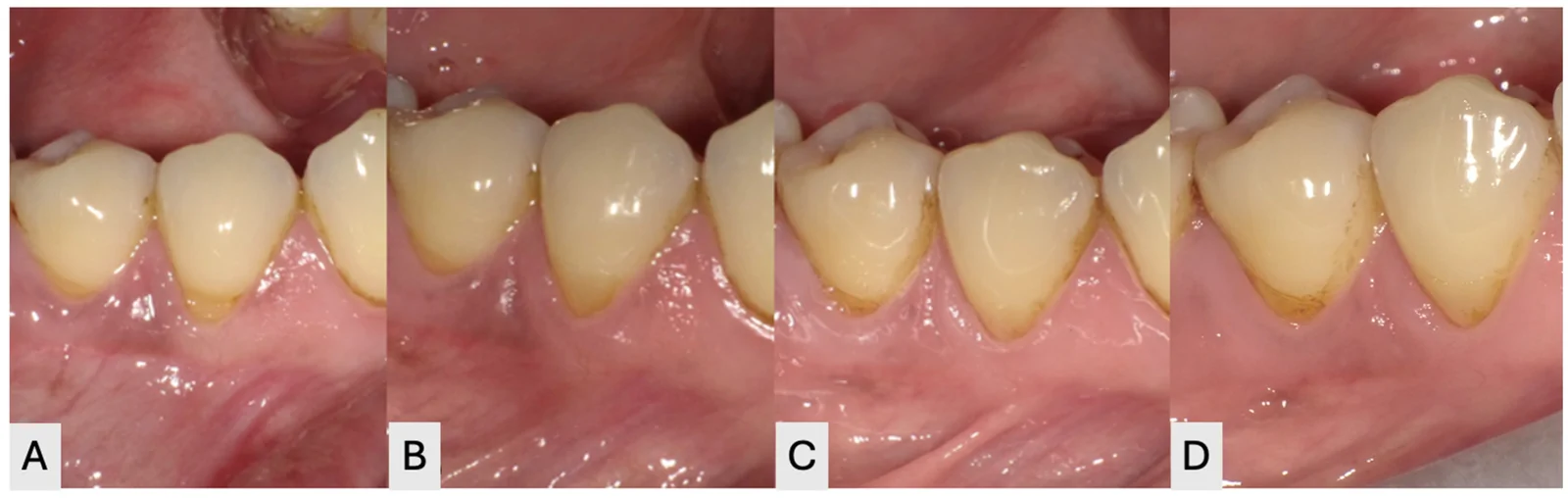
Clinical image of FS at (**A**) baseline, (**B**) 18 months, (**C**) 48 months, and (**D**) 72 months.

**Figure 4:**
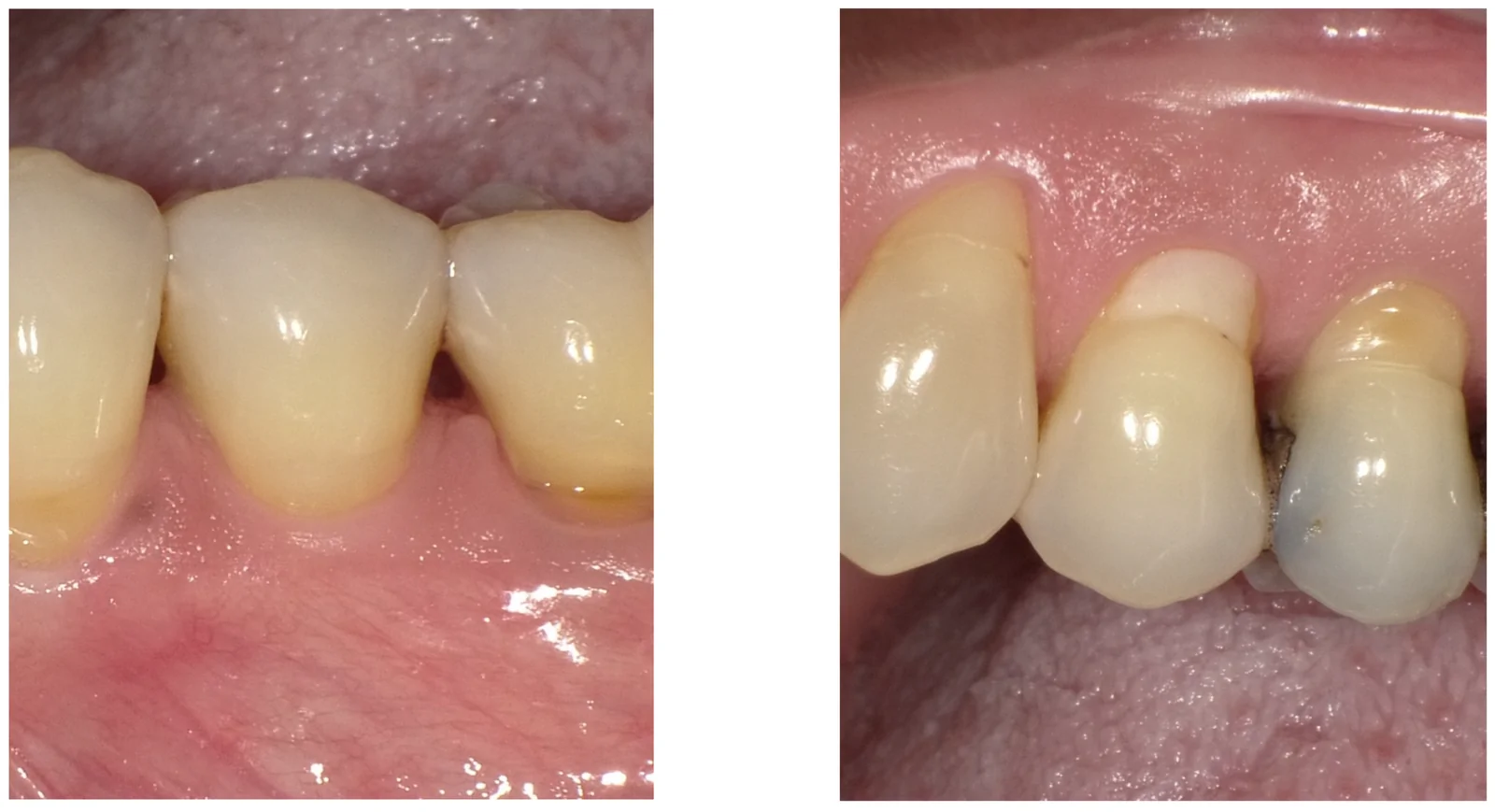
Clinical image of BL (**left**) and FS (**right**) at 72 months.

**Figure 5:**
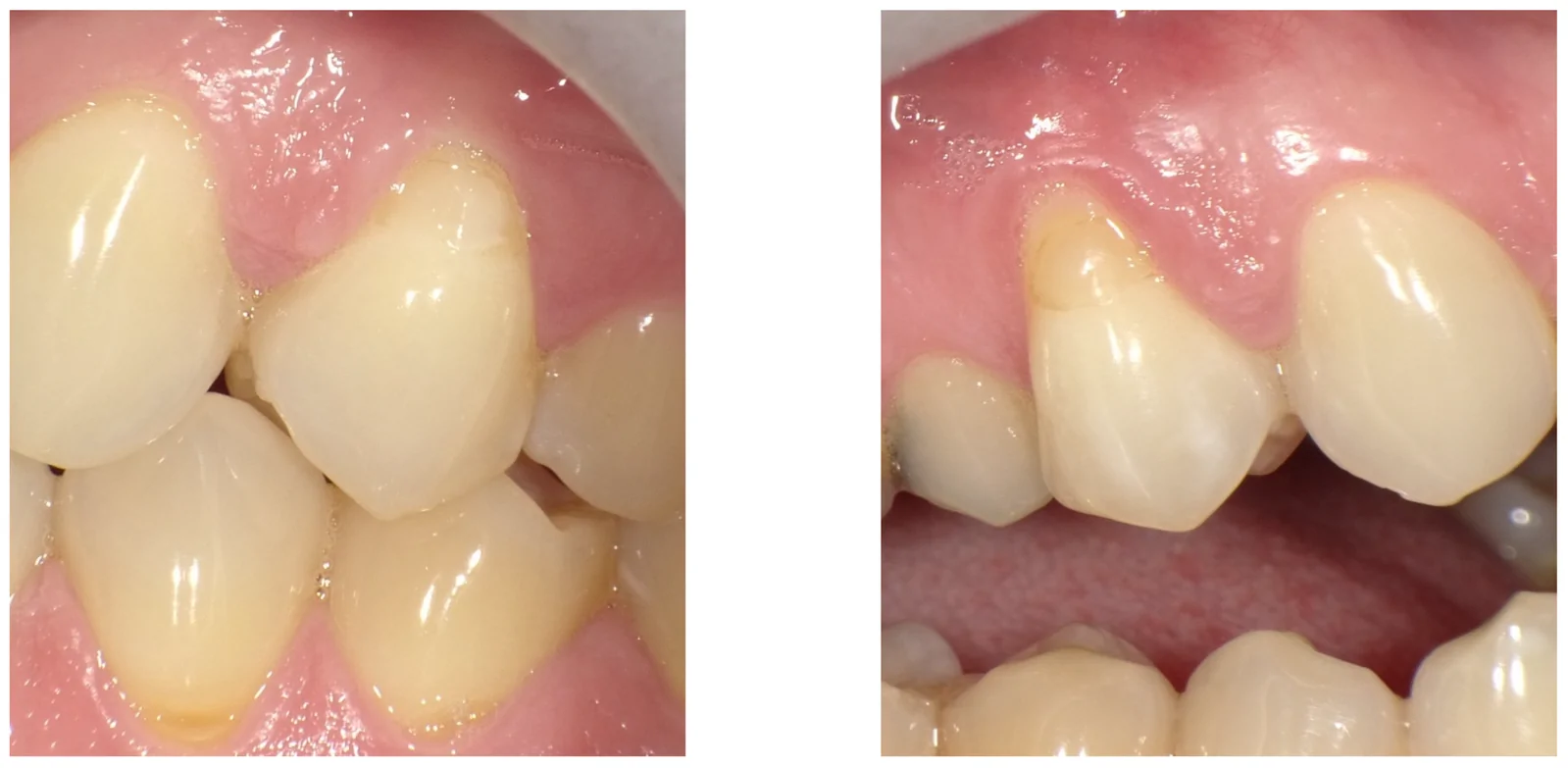
Clinical image of BL (**left**) and FS (**right**) at 72 months.

Clinical evaluations under Hickel Criteria (Table S1) showed similar performances by both material systems compared under the scale of 1 being Clinically excellent/very good to 5 being Clinically poor (replacement necessary). The list of Hickel Scores is presented in Table S2. When comparing the number of cases that scored a Clinically Excellent/Very Good rating, both restoration systems performed fairly evenly as well. Most restorations were rated clinically excellent/very good (score 1) across categories; for example, postoperative sensitivity was scored 1 in 95.5% of restorations in each group (maximum score 2), and tooth integrity was scored 1 in 90.9% of each group (maximum score 2). Recurrent caries (Hickel score ≥2) was, however, recorded in 3 BL restorations (scores 2, 2, and 4) and 2 FS restorations (scores 2 and 4); one participant exhibited cavitated caries (score 4) on both restorations. No restoration was lost in either group. No relevant differences were observed between restorative systems in the remaining evaluated categories. Representative clinical photographs taken for both BL and FS at each visit are presented in Figure 2 and Figure 3, and Figure 4 and Figure 5 also include a comparison at 72 months.

## 4. Discussion

This 72-month randomized, split-mouth clinical trial compared the long-term clinical performance of a giomer-based restorative material (BEAUTIFIL II LS) and a nanohybrid restorative composite (Filtek Supreme Universal) in Class V non-carious cervical lesions. Among the evaluable restorations at 72 months, no restoration was lost, and no statistically significant difference was detected between the two materials across the esthetic, functional, and biological domains of the modified Hickel/FDI criteria. Because long-term attrition reduced the statistical power available at this time point, these results are interpreted as evidence of similar observed clinical performance rather than as formal equivalence.

These findings are consistent with the earlier reports from the same trial series. At the 18-month follow-up, both materials performed comparably across the modified Hickel/FDI domains [11], and at 48 months no statistically significant differences were observed between BEAUTIFIL II LS and Filtek Supreme Universal [13]. The present 72-month data extend this pattern by a further 24 months, indicating that the comparable clinical behavior of the two materials persisted over the extended follow-up, while acknowledging that progressively fewer participants were available for evaluation at each successive recall.

BEAUTIFIL II LS contains surface pre-reacted glass-ionomer (S-PRG) filler, which can release multiple ions and has been reported to provide bioactive properties such as acid neutralization, fluoride release, and a remineralizing potential [9,14]. In principle, these properties, together with the lower polymerization shrinkage of the material, might be expected to confer a clinical advantage in the cervical environment. However, recurrent caries was recorded in both groups (3 BL and 2 FS restorations, including one participant with cavitated lesions on both sides), so any caries-preventive contribution of the S-PRG filler was not sufficient to prevent secondary caries in this cohort. The ion-release properties should therefore be regarded as a theoretical rather than a demonstrated clinical benefit in this specific application.

From a clinical perspective, both BEAUTIFIL II LS and Filtek Supreme Universal can be considered acceptable options for the direct restoration of Class V non-carious cervical lesions over the long term. In the absence of a detectable difference in clinical performance, material selection may reasonably be guided by case-specific requirements, handling characteristics, esthetic demands, cost, and operator familiarity, rather than by an assumed superiority of either filler technology.

Several limitations should be considered when interpreting these results. The principal limitation is participant attrition: of the 49 participants enrolled, 26 attended the 72-month visit and 19 contributed complete paired observations, which reduced the statistical power available to detect small between-material differences and introduced the possibility of selection bias. Accordingly, an available-case approach was used and a prespecified sensitivity analysis restricted to complete pairs was performed; non-significant comparisons are therefore interpreted cautiously and should not be read as proof of equivalence. In addition, the trial evaluated one specific pair of materials and adhesive protocols, so the findings cannot be generalized to other giomer-based or nanohybrid restorative systems that differ in formulation, filler content, or handling. Finally, because the etiology of non-carious cervical lesions is multifactorial and associated with occlusal stress, toothbrushing trauma [15,16], and progressive marginal change over time [17], durable long-term outcomes depend not only on material selection but also on the management of these underlying risk factors.

## 5. Conclusions

Within the limitations of this 72-month randomized split-mouth clinical trial, BEAUTIFIL II LS and Filtek Supreme Universal showed similar clinical behavior when used for direct restoration of Class V non-carious cervical lesions. Both materials may be considered acceptable restorative options, and material selection may be guided by clinical requirements, handling characteristics, esthetic demands, and clinician preference. Future studies with larger retained samples are warranted to confirm these long-term findings.

## Figures and Tables

**Table 1:** Socio-demographic data of the patients included in the study at the 72-month follow-up.

Participants	*n* = 26
Sex	
Male	12
Female	14
Age (years), Mean ± Standard Deviation	62.39 ± 14.86
Smoker	5
Ethnicity	
Native American	0
Hispanic/Latino	3
Asian	4
Black	6
White	10
Prefer Not to Answer	3

## Data Availability

Data supporting the findings of this study are not publicly available due to privacy concerns. Further details about the data and conditions for access can be obtained from the corresponding author upon reasonable request.

## Supplementary Materials

The following supporting information can be downloaded at: https://www.mdpi.com/article/10.3390/dj14090603/s1. Table S1: Modified Hickel/FDI scoring criteria; Table S2: Distribution of Hickel/FDI scores by restorative material at the 72-month evaluation.
